# Overexpression of the *GmERF071* gene confers resistance to soybean cyst nematode in soybean

**DOI:** 10.1002/tpg2.70033

**Published:** 2025-04-29

**Authors:** Erhui Xiong, Jiaqi Xu, Pingzhang Feng, Yun Lian, Xiaoling Zhi, Ke Li, Erhan Zhang, Bing Li, Shijie Zhao, Changzhong Liu, Chengyu Wei, Panpan Li, Yaping Zhao, Lipei Zhao, Mengwei Zheng, Heng Zhang, Yi Li, Shanshan Chu, Yongqing Jiao

**Affiliations:** ^1^ Collaborative Innovation Center of Henan Grain Crops, College of Agronomy Henan Agricultural University Zhengzhou China; ^2^ Institute of Crop Molecular Breeding Henan Academy of Agricultural Sciences Zhengzhou China

## Abstract

Soybean cyst nematode (SCN) is one of the most harmful pests, causing major reductions in soybean yield globally. The validation and functional characterization of SCN resistance genes are crucial to improving soybean yield worldwide. Herein, we describe an SCN resistance gene, *GmERF071* (*Glyma.19g262700*). *GmERF071* is a hydrophilic, unstable protein with an AP2/ERF subfamily ethylene response transcription factor domain, which is localized in the nucleus. Overexpression of *GmERF071* enhanced SCN resistance in the soybean stable genetic transformation and root systems. RNA‐seq analysis revealed 394 upregulated and 132 downregulated differentially expressed genes (DEGs) in *GmERF071* overexpression transgenic plants. The DEGs participated in plant‐pathogen interactions, mitogen‐activated protein kinase signaling, plant hormone signal transduction, response to chitin, response to carbohydrates, response to wounding in starch and sucrose metabolism, phenylpropionic acid biosynthesis, and flavonoid biosynthesis. Nine candidate DEGs were verified using real‐time quantitative reverse transcription PCR. These results suggest that *GmERF071* plays a key role in SCN resistance and could be used in genomics‐assisted breeding to develop soybean varieties with increased resistance to SCN.

## Core Ideas

- A novel soybean cyst nematode (SCN) resistance gene *GmERF071* was identified.
- Overexpression of *GmERF071* effectively enhanced the SCN resistance of soybeans.
- RNA‐seq results suggested that *GmERF071* plays a key role in SCN resistance.
- GmERF071 affects the growth and development of SCN.

**Abbreviations**
DEGs: differentially expressed genes
ERF: ethylene response transcription factor
GFP: green fluorescent protein
GO: gene ontology
KEGG: Kyoto Encyclopedia of Genes and Genomes
MAPK: mitogen‐activated protein kinase
SCN: soybean cyst nematode
SIM: shoot induction medium

## 1 INTRODUCTION

Soybean (*Glycine max*) is a vital source of food, protein, and oil worldwide (Hu et al., 2024). As one of the main oil crops in China, soybeans play an irreplaceable role in food security and the national economy (Fares, 2023; Lyu & Sun, 2021). The soybean cyst nematode (SCN) is one of the most harmful pests and diseases of soybean plants (Peng et al., 2021). In the United States, the annual output value loss of soybeans caused by SCN is >$1 billion (Bandara et al., 2020). In the main soybean‐producing areas of China, SCN has caused >30% soybean yield reduction (Duan et al., 2011). Using molecular biology to develop resistant soybean varieties is one of the most effective means of controlling SCN.

To improve the capability and precision of breeding paths, various studies have focused on identifying the genes controlling nematode resistance (Kadam et al., 2016; Gou et al., 2023; Guo, 2020). Currently, *Rhg1* and *Rhg4* are the two most important SCN resistance loci (Concibido et al., 2004; Cook et al., 2012; Liu et al., 2012). Whole‐genome re‐sequencing reveals the impact of the interaction of copy number variants of the *rhg1* and *Rhg4* genes on broad‐based resistance to SCN (Patil et al., 2019). Among the soybean varieties with SCN resistance, 90% were PI88788, Peking (PI548402), PI209332, and PI437654 (Usovsky et al., 2021). SCN resistance in PI88788 requires the *Rhg1‐b* allele, whereas SCN resistance in Peking requires the *Rhg1‐a* and *Rhg4* alleles (Huang et al., 2019a). The *rhg1‐a* (*Rhg1* low‐copy) nematode resistance source harbors a copia‐family retrotransposon within the Rhg1‐encoded α‐SNAP gene (Bayless et al., 2019). In addition, soybean *rhg1*, which has provided durable disease resistance for a few decades, is gradually being defeated due to pathogen evolution (Bent, 2022). None of the four different *Rhg1*‐encoded genes—*Glyma.18G022400*, *Glyma.18G022500*, *Glyma.18G022600*, and *Glyma.18G022700*—have similarities to previously identified resistance genes. However, the α‐SNAP gene *Glyma.18G022500* has amino acid polymorphisms relative to the wild‐type *Rhg1* gene alleles present in SCN‐susceptible soybeans (Cook et al., 2012). Quantitative trait loci located on chromosome 18 (*rhg1‐a*) and chromosome 11 (*rhg2*) were determined to confer SCN resistance in PI 90763, and the *rhg2* was fine‐mapped to a 169‐kbp region pinpointing GmSNAP11 as the strongest candidate gene (Basnet et al., 2022). Simultaneous application of diverse partially operative SCN controls may be both essential and durable. Identifying novel genetic resources involved in susceptibility and resistance mechanisms provides a promising approach for the developing novel SCN‐resistant cultivars. The loss‐of‐function mutations of *GmSNAP02* confer resistance to nematodes in soybean (Usovsky et al., 2023), and *cqSCN‐006* alters the expression of a γ‐SNAP protein participating in susceptible responses to SCN (Bent, 2022). Genome reorganization of the GmSHMT gene family in soybeans shows a lack of functional redundancy in resistance to SCN (Lakhssassi et al., 2019). SCN resistance requires both the *GmSHMT08* (at the *Rhg4* locus) and *GmSNAP18* in Peking‐type resistance (Lakhssassi et al., 2020). Proteomic and transcriptomic analyses revealed the involvement of both THF and PLP sites at *GmSHMT08* in SCN resistance (Lakhssassi et al., 2022). Furthermore. The *GmSHMT08* plays an important role in *beet cyst nematode* (L. P. Zhang et al., 2023; Zhao et al., 2022). Additionally, genes such as *GmSNAP11* (Lakhssassi et al., 2017), *HgSLP‐1* (Bekal et al., 2016), and *GmSAMT1* (Lin et al., 2016) confer resistance to SCN.

In our previous study, the transcriptomes of PI88788‐ and Peking‐type soybean accessions resistant to SCN were compared, and an upregulated expression of *GmERF071* encoding ethylene response transcription factor (ERF) was found in both sources (Chu et al., 2022). *GmERF071* has high homology with maize and *Arabidopsis*, which contains AP2 domain and cis‐acting elements involved in stress response (Wang et al., 2023). In *Arabidopsis*, the *AtERF71/HRE2/RAP2.2* is involved in abiotic stress, such as hypoxia (Giuntoli & Perata, 2018; Schippers et al., 2024) and salt stress (Seok et al., 2022), and biotic stress, such as *Botrytis cinerea* infection (Zhao et al., 2012). In rice, overexpression of the *OsERF71* transcription factor alters rice root structure and drought resistance (Ahn et al., 2017; Lee et al., 2016, 2017; J. J. Li et al., 2018). A few genes of the *GmERF* family, such as *GmERF5* and *GmERF113*, are involved in soybean resistance to *Phytophthora sojae* (Dong et al., 2015; Zhao et al., 2017). In addition, *ZmERF105* positively regulates the resistance response of maize to *Exserohilum turcicum* (Z. Y. Zang et al., 2020), and *AtERF19* negatively regulates pattern‐triggered immunity against *Botrytis cinerea* and Pseudomonas syringae (Huang et al., 2019). Tomato *ERF2* may directly or indirectly regulate Pto, PR1b1, and PR‐P2 expression and enhance tomato resistance to *Septoria. lycopersici* (Yang et al., 2021). To date, limited research is available on the role of *GmERF071* in the SCN resistance. Thus, we aimed to verify the molecular function of *GmERF071* in the SCN resistance of soybeans. The results of this study will help elucidate the mechanism of action of *GmERF071* in the defense response of soybeans to SCN.

## 2 MATERIALS AND METHODS

### 2.1 Phylogenetic analysis of *GmERF071*

The accession number *GmERF071* was obtained from the public plant RNA‐seq database, including RNA‐seq data of *Arabidopsis*, maize, rice, soybean, wheat, and cotton. A phylogenetic tree was constructed using the MEGA 5.1 software (www.megasoftware.net) and the maximum‐likelihood method (Hall, 2013; Tao et al., 2019). The protein sequence was further compared with those of *Arabidopsis* and other crops with high homology.

### 2.2 Bioinformatics analysis of *GmERF071*

The 3000 bp sequence before the transcription starting site of *GmERF071* was subjected to reverse complementation in Primer5.0 and searched for cis‐acting regulatory elements across the PlantCARE database (http://bioinformatics.psb.ugent.be/webtools/plantcare/html) (Lescot et al., 2002). The physical and chemical properties of *GmERF071* were analyzed using ProtParam (https://web.expasy.org/protparam/). Multiple sequence alignments of the expressed sequence tags of *GmERF071* were performed using ClustalX (ver. 1.83). The secondary structure of the coding protein of GmERF071 was predicted using SOPMA (https://npsa.lyon.cserm.fr/cgi‐bin/npsa_automat.pl?page=/NPSA/npsa_sopma.html) (Geourjon & Deleage, 1995), and the tertiary structure of the protein was modeled online using SWISS‐MODEL (https://swissmodel.expasy.org/) (Waterhouse et al., 2018).

**Core Ideas**

- A novel soybean cyst nematode (SCN) resistance gene *GmERF071* was identified.
- Overexpression of *GmERF071* effectively enhanced the SCN resistance of soybeans.
- RNA‐seq results suggested that *GmERF071* plays a key role in SCN resistance.
- GmERF071 affects the growth and development of SCN.

### 2.3 Subcellular localization of the GmERF071 protein

A recombinant plasmid containing 35S‐green fluorescent protein (GFP) was transformed into *Agrobacterium* GV3101 and identified using PCR. The fusion vector (35S::*GmERF071*‐GFP) and control (35S‐GFP) were infiltrated into tobacco (*Nicotiana benthamiana*) leaf epidermal cells for instantaneous expression. The fluorescence signal of the tobacco leaves was observed using a laser confocal microscope (Leica).

### 2.4 Genetic transformation of *GmERF071* into soybean

Soybean Tianlong 1, which is susceptible to SCN and Race 3 SCN (*Heterodera glycines*), was provided by the Henan Academy of Agricultural Sciences. Total RNA was extracted from soybeans using TRIzol Universal Total RNA Extraction Reagent (TianGen Biotechnology). Reverse transcription was conducted using a Reverse Transcription Kit (Vazyme Biotech Co.), and *GmERF071* was amplified using PCR with cDNA as a template. The purified gel extraction product containing the *GmERF071* DNA fragment was recovered and cloned into the cloning vector PJL12 with CaMV 35S promoter.

The genetic transformation of *GmERF071* was performed following methods used for the genetic transformation of *Agrobacterium rhizogenes*, with an empty vector as control. Soybean seeds were sterilized with chlorine gas for 12–14 h and then germinated in Hogland medium for 4 days under 16‐ /8‐h light/dark conditions in a growth chamber at 25–26°C. Germinating seedlings were used for hairy root transformation following methods described previously for *A. rhizogenes* strain K599 and then analyzed (Kereszt et al., 2007).

Our lab has a stable soybean genetic transformation system. In brief, the germinated seeds were cut in half, the seed coats were removed, and the hypocotyl was retained close to the cotyledons, approximately at 3–5 mm. A few lines near the growth point of the cotyledons were lightly scratched. The seeds were soaked in an *Agrobacterium* suspension for 30 min, dried on sterile filter paper, placed on solid CCM medium with sterile filter paper, and then incubated in the dark for 3 days. Then, the newly grown hypocotyls were cut off again, leaving only approximately 3–5 mm of the hypocotyl near the cotyledons, and scrape off the shoot buds near the growth point were scraped off. The samples were washed with sterile water and liquid shoot induction medium (SIM) and placed in a solid SIM medium without a selection agent. After selection, the cotyledons of the regenerated buds were removed and placed in the shoot elongation medium, subculturing once a week. After growing to 3–5 cm, the regenerated shoots were cut off and inserted into the rooting medium to induce root formation. The GeneTure BAR Test Kit (Artron) for the *Bar* gene and the herbicide Basta were used to identify the transformed soybean. Finally, RT‐qPCR was performed to determine the expression of *GmERF071* in transgenic soybean and Tianlong 1. T2 homozygous transgenic lines were used for further research.

### 2.5 Identification of SCN resistance of transgenic plants

*Heterodera glycines* race 3, provided by the Henan Academy of Agricultural Science, was bred with the susceptible soybean variety (Lee). After washing the root and sieving, cysts were collected using a 60‐mesh sieve (Acedo & Dropkin, 1982). When the true leaves of Tianlong 1 and *GmERF071* overexpression plants were fully unfolded, the cysts were vaccinated. After 25 days of plants inoculation at 25–28°C with 14 h/day illumination, cysts were collected and counted.

Further, Tianlong 1 and *GmERF071* overexpression plants were infected by cysts, and then the NaOCl‐acid fuchsin‐glycerin staining technique was used for nematode staining. Briefly, the roots were washed under running water, cut into 1–2 cm segments, added with 20 mL of 5.25% NaOCl for 4 min, washed under running water, heated and boiled for 30 s with acid fuchsin staining, decolorized with acidified glycerin, and then observed under a microscope.

### 2.6 RNA extraction and sequencing

The total RNA of the transgenic soybean (T2 homozygous transgenic lines) and control roots at the V1 growth stage were retrieved using the TRIzol reagent (Yao et al., 2023);. The purity of the RNA samples was determined using a NanoDrop ND‐1000 microspectrophotometer (Thermo Fisher) and an Agilent 2100 bioanalyzer (Agilent Technologies). Transcriptome sequencing was performed by Biomarker Technology Co. Ltd. Three biological replicates were used in each experiment. After cDNA library construction, the quality of the retrieved cDNA was tested using the Qsep400 method and sequenced on an Illumina MiSeq with PE 150 mode. The raw data were preprocessed, and the reads were mapped to reference sequences (https://www.soybase.org/) and converted into fragments per kilobase of transcript per million fragments mapped using StringTie.

### 2.7 Identification of differentially expressed genes (DEGs) and functional annotation

A pairwise transcriptome comparison between the transgenic soybean and control groups was used to identify DEGs between the two groups. DEGs with |log_2_FC (fold‐change) | > 1 were selected. Gene ontology (GO) enrichment analyses for these DEGs were carried out using the “enrichGO” function of the clusterProfiler package (Yu et al., 2012) with a *p*‐value < 0.05. Kyoto Encyclopedia of Genes and Genomes (KEGG) pathway enrichment analysis (https://www.genome.jp/kegg/) was performed using KOBAS software (http://kobas.cbi.pku.edu.cn/index.php). The clusterProfiler 4.0 package was used to identify and describe GO terms and KEGG pathways enriched by the DEGs.

### 2.8 Gene expression analysis

Total RNA was extracted from root, stem, leaf, anther, leaf sheath, and booting spike tissues using TRIzoL reagent (Invitrogen). qRT‐PCR was performed using SYBR Premix Ex Taq II (TaKaRa Bio) with the CFX96 Real‐Time PCR Detection System (Bio‐Rad). The qRT‐PCR of the nine candidate genes was conducted using *actin* of *Glyma.18G290800* as a reference. The primers used are listed in Table S1.

### 2.9 Statistical data analysis

Quantitative data were processed using SPSS (version 22.0; IBM). Differences among groups were compared using one‐way analysis of variance or the *t*‐test, when applicable. Statistical differences are marked with different capitalized (*p *< 0.01) or lowercase (*p *< 0.05) letters.

## 3 RESULTS

### 3.1 Phylogenetic analysis, physical, and chemical properties of *GmERF071*

**FIGURE 1:**
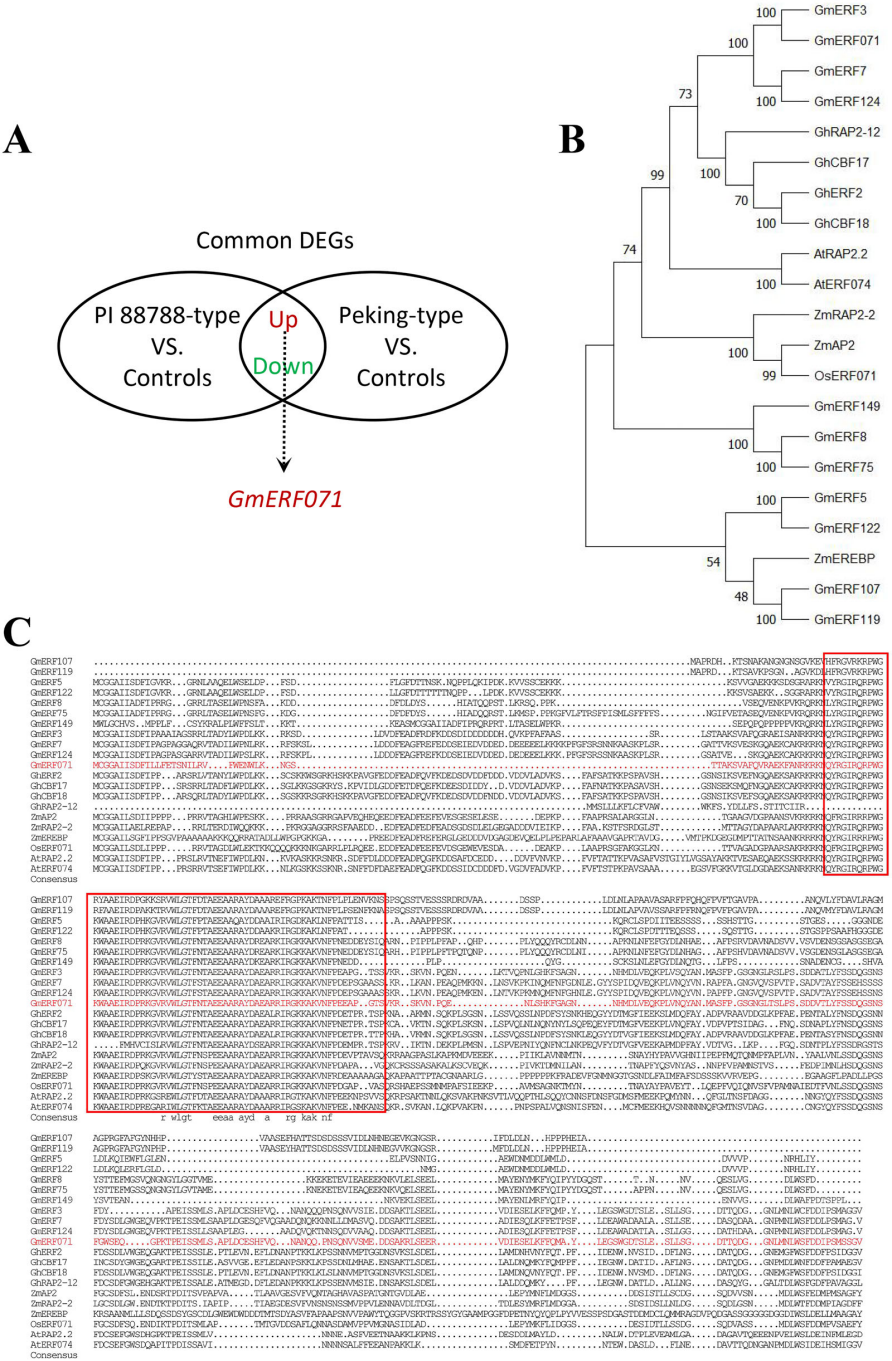
Bioinformatics analysis of *GmERF071*. (A) *GmERF071* was upregulated in Peking‐type and PI88788‐type resistant sources; (B) Phylogenetic analysis of *GmERF071*; (C) Multiple amino acid sequence comparison of *GmERF071*. The red box is the AP2 domain. DEGs, differentially expressed genes.

To provide novel insights into the molecular biology of soybean varieties resistant to SCN, our previous study showed that the 77 genes were upregulated between PI 88788‐ and Peking‐type resistant sources; among them, only one gene of *GmERF71* is a transcription factor, which belongs to ethylene‐response‐factor family (Figure 1A) (Chu et al., 2022). Phylogenetic analysis and sequence alignment indicated that the amino acid sequence of GmERF071 shares high homology with GmERF3, GmERF7, and GmERF124 in soybean; AtAP2 and AtERF074 in *Arabidopsis*; ZmAP2 and ZmRAP2‐2 in maize; and OsERF071 in rice (Figure 1B,C). GmERF071 is a hydrophilic and unstable protein (Figure S1A). Protein domain structure prediction analysis indicated that *GmERF071* is a transcription factor containing an AP2/ERF domain (Figure S1B). The prognosis of *cis*‐acting elements in the promoter of *GmERF071* showed that *GmERF071* could respond to the induction of various hormones and the soybean defense system induced by light stress, drought stress, salt stress, and other stresses.

### 3.2 Expression and subcellular localization of GmERF071

**FIGURE 2:**
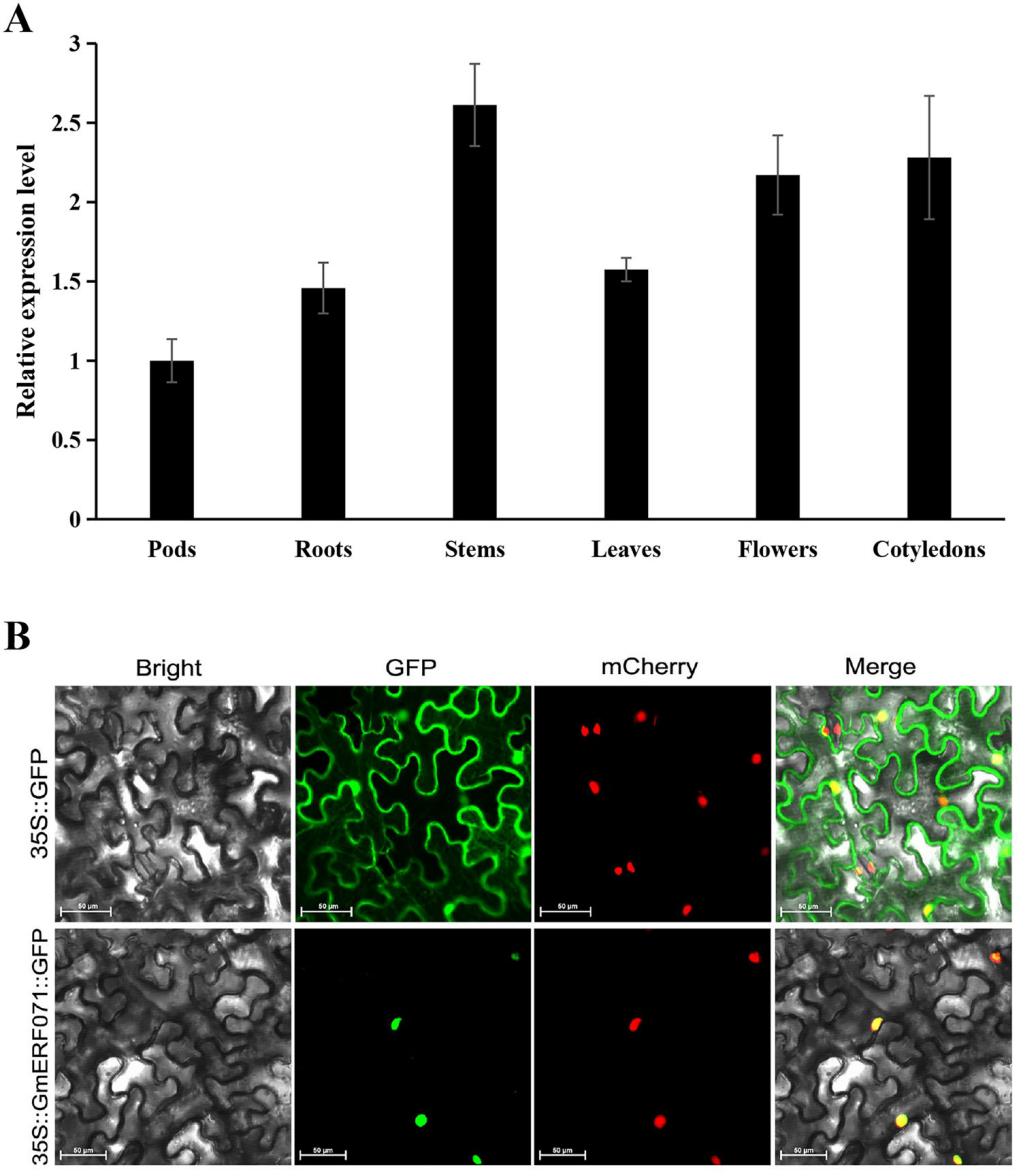
Expression and subcellular localization of GmERF071. (A) The relative expression of GmERF071 in different tissue; (B) subcellular localization of *GmERF071* transiently expressed in the nucleus of tobacco leaf cells. 35S::GFP as control, 35S::GmERF071::GFP located to nucleus. mCherry, nuclear mark. Bar, 50 µm.

To analyze the expression of *GmERF071*, we performed qRT‐PCR using total RNA isolated from various organs of Tianlong 1, including roots, stems, leaves, flowers, pods, and cotyledons. *GmERF071* transcript was most abundant in stems, followed by cotyledons, flowers, then leaves, roots, and pods (Figure 2A); the relative expression levels of *GmERF071* in different tissues were not huge. The *GmERF071* gene was cloned into the overexpressed vector 35S::*GmERF071*‐GFP and transformed into *Agrobacterium* GV3101. Subcellular localization of GmERF071 indicated that GFP signaling was concentrated in the nuclei of tobacco leaf cells (Figure 2B).

### 3.3 Overexpression of *GmERF071* enhances soybean resistance to cyst nematode

**FIGURE 3:**
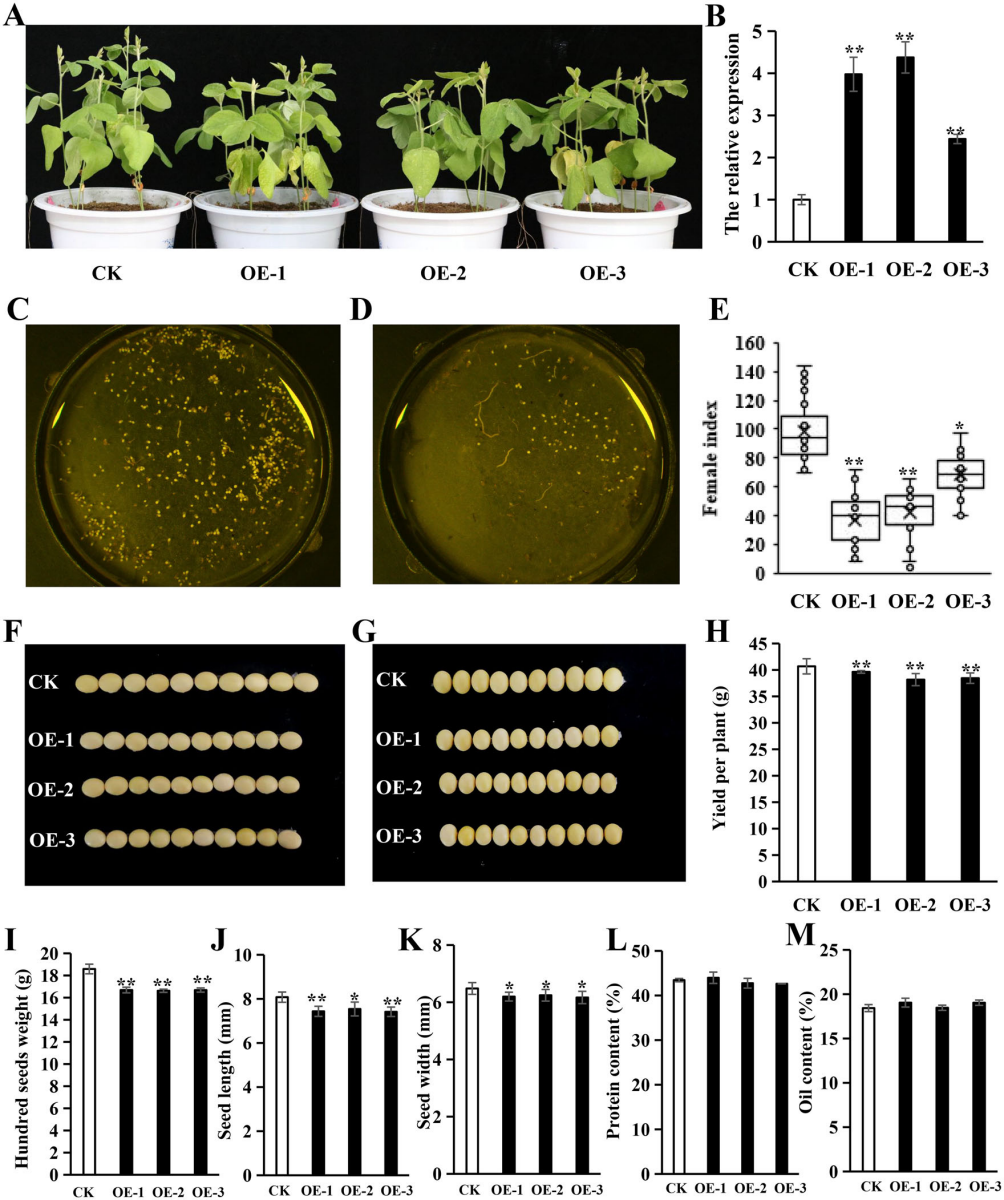
Phenotypic analysis of *GmERF071* overexpression transgenic plants. (A) Phenotype of CK and overexpression transgenic lines; (B) Relative expression of three overexpression transgenic lines; (C) Number of cysts identified from the root of Tianlong 1, a reference background material. Bar = 3 cm. (D) Number of cysts identified from the root of *GmERF071* transgenic strain. Bar = 3 cm. (E) FI map for insect resistance identification of CK and *GmERF071* transgenic plants. (F–G) Comparison of seed length (F) and seed width (G) between control and transgenic plant seeds. Bar = 1 cm; (H–L) Data histogram of 100‐seeds weight (H), seed length (I), seed width (J), protein content (K), and oil content (L) between control and transgenic lines. (**p < 0.01; *p < 0.05).

The genetic transformation of *GmERF071* was performed following methods used for the genetic transformation of *A. rhizogenes*. The cyst number on the transgenic hairy roots overexpressing GmERF071 was significantly decreased compared with that on the control, suggesting that *GmERF071* was involved in resistance to SCN (Figure S2). Furthermore, soybean stable genetic transformation was used to verify the function of *GmERF071* in SCN resistance, and *GmERF071* overexpressing transgenic plants were obtained. Three independent lines of transgenic plants were screened using qRT‐PCR, and then T2 transgenic lines were obtained (Figure 3A,B). Cyst counting results showed that the Female Index (FI) of the transgenic strain (FI ranged from 50 to 60, moderately resistant) was much lower than that of the control Tianlong 1 (FI was approximately 100, susceptible) (Figure 3C–E). Further, we observed the SCN penetration and development in Tianlong 1 and *GmERF071* transgenic lines after infecting cysts, and the growth and development rate of SCN in the *GmERF071* transgenic lines was significantly slower than that in Tianlong 1 (Figure S3). These results revealed that *GmERF071* effectively enhanced SCN resistance of soybeans.

### 3.4 Phenotypes of *GmERF071* overexpression transgenic plants

Seed traits of the *GmERF071* transgenic plants were investigated. The average plant height of *GmERF071* transgenic lines at vegetative growth stage 2 was significantly lower than that of Tianlong 1. Seed length, seed width, 100‐seed weight, and yield per plant of seed were significantly decreased in *GmERF071* transgenic plants (Figure 3F–K), whereas oil content and protein content were not significantly affected (Figure 3L,M).

### 3.5 Transcriptome analysis of *GmERF071* overexpression transgenic plants

**FIGURE 4:**
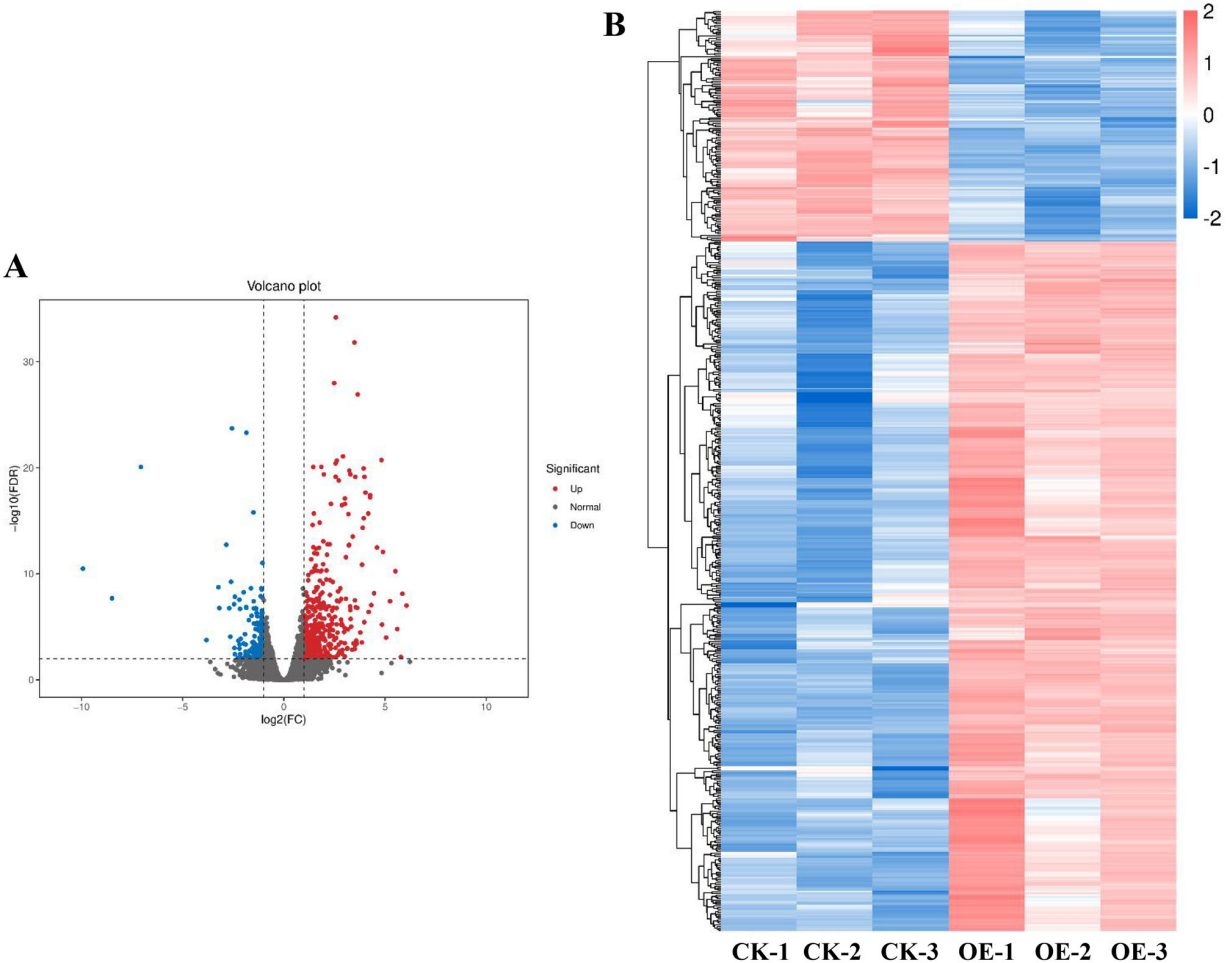
Identification of differentially expressed genes (DEGs). (A) Volcano plot displayed the distribution of DEGs. The *x*‐axis represents the log_2_FC of gene expression, and the *y*‐axis represents the statistical significance of the differential expression analysis performed between the two groups. FC, fold change; FDR, false discovery rate. (B) Heatmap of DEGs.

Transcriptome analysis was performed after RNA extraction from the roots of Tianlong 1 and *GmERF071* transgenic plants with no SCN treatment. The results revealed 526 DEGs, including 394 upregulated and 132 downregulated DEGs (Figure 4A,B). The 10 most upregulated DEGs, MYB/HD‐like transcription factor (*Glyma.12G104800* and *Glyma.06G300200*), DUF4228 domain‐containing protein (*Glyma.18G118300*), and F‐box protein (*Glyma.17G011700*), as well as the 10 most downregulated DEGs, AAA + ATPase domain‐containing protein (*Glyma.18G254100*), leucine‐rich repeat‐containing (*Glyma.16G174500*), and Protein E6 (*Glyma.15G077600*), are listed in Table S2.

**FIGURE 5:**
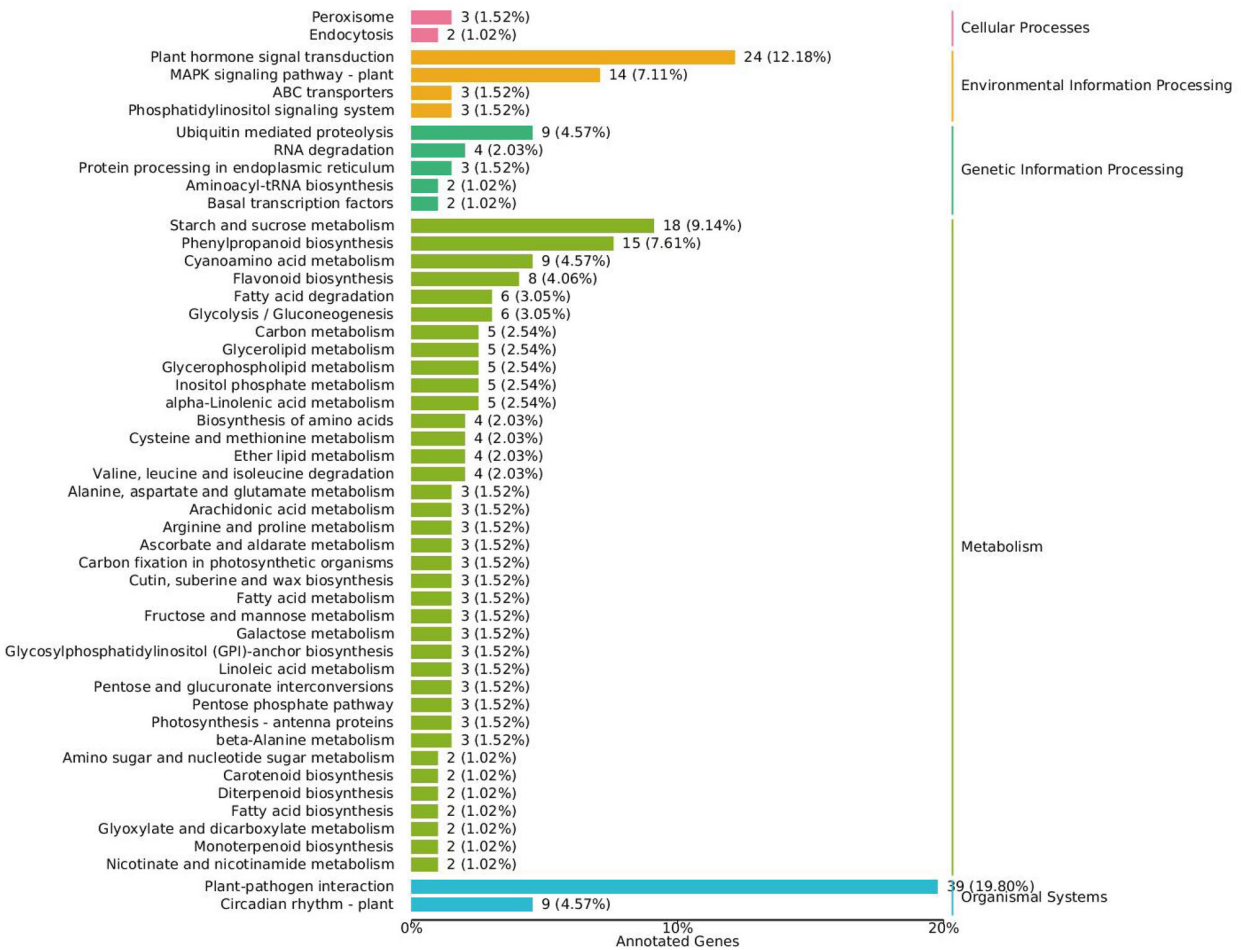
Kyoto Encyclopedia of Genes and Genomes (KEGG) classification of differently expressed genes (DEGs).

**FIGURE 6:**
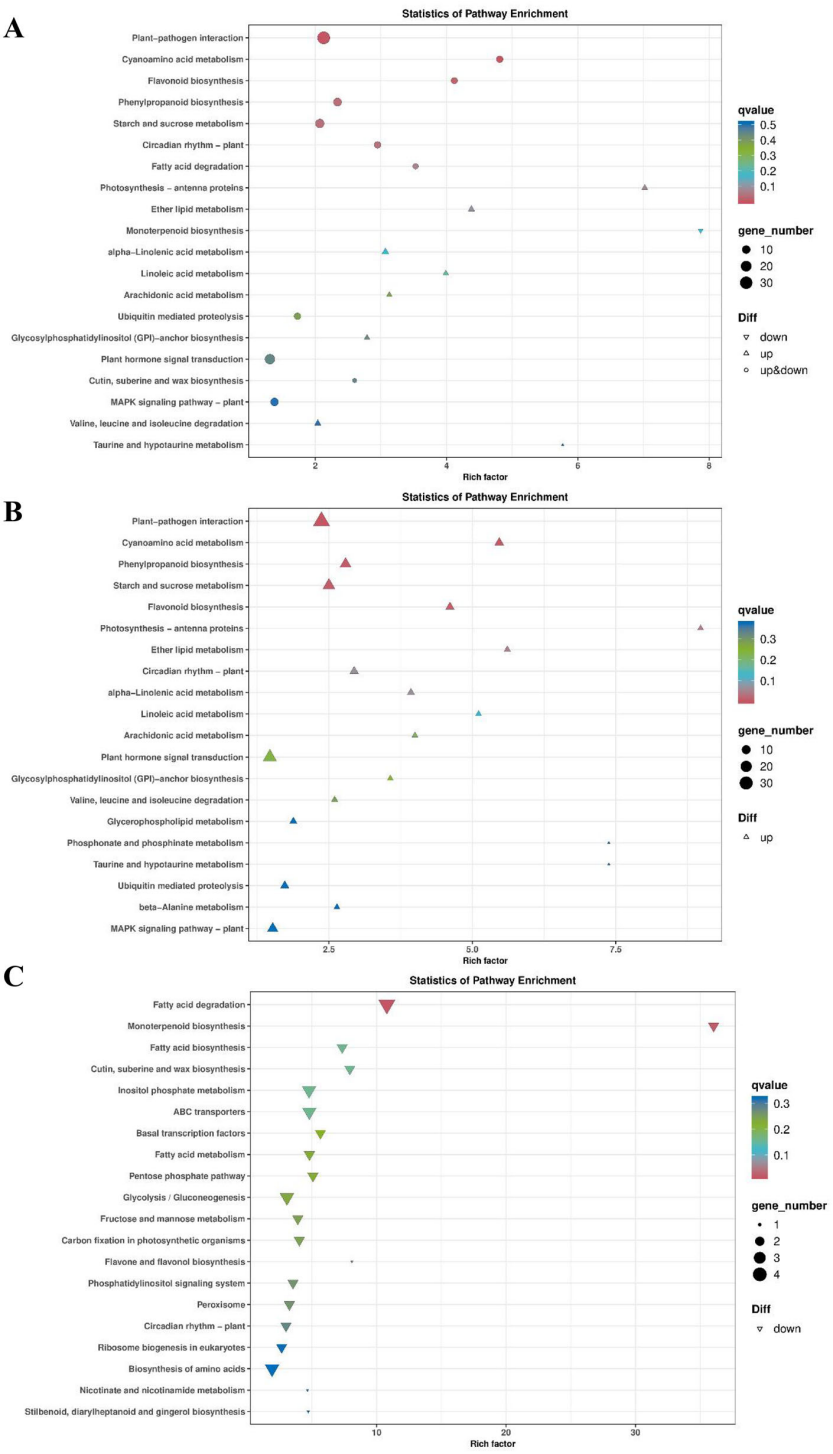
Kyoto Encyclopedia of Genes and Genomes (KEGG) pathway enrichment results of DEGs. (A) KEGG pathway enrichment of DEGs; (B) KEGG pathway enrichment of upregulated DEGs; (C) KEGG pathway enrichment of down‐regulated DEGs.

KEGG analysis was performed to further investigate the pathways associated with the DEGs. The results of KEGG classification suggested that the DEGs were mostly divided into plant hormone signal transduction, plant‐pathogen interactions of organismal systems, the mitogen‐activated protein kinase (MAPK) signaling pathway of environmental information processing, starch and sucrose metabolism, and phenylpropanoid biosynthesis of metabolism in the KEGG classification (Figure 5). In addition, the KEGG pathway was enriched in plant‐pathogen interactions, starch and sucrose metabolism, phenylpropanoid biosynthesis, the MAPK signaling pathway, and plant hormone signal transduction (Figure 6A). Furthermore, the upregulated DEGs were enriched in starch and sucrose metabolism, plant‐pathogen interactions, cyanoamino acid metabolism, plant hormone signal transduction, phenylpropanoid biosynthesis, MAPK signaling pathway, and flavonoid biosynthesis (Figure 6B), whereas the downregulated DEGs were enriched in fatty acid degradation, monoterpenoid biosynthesis, ABC transporters, and inositol phosphate metabolism (Figure 6C). These findings suggest that GmERF071 plays a key role in SCN resistance by regulating starch and sucrose metabolism, plant‐pathogen interactions, and plant hormone signal transduction pathways.

### 3.6 Validation of candidate DEGs

**FIGURE 7:**
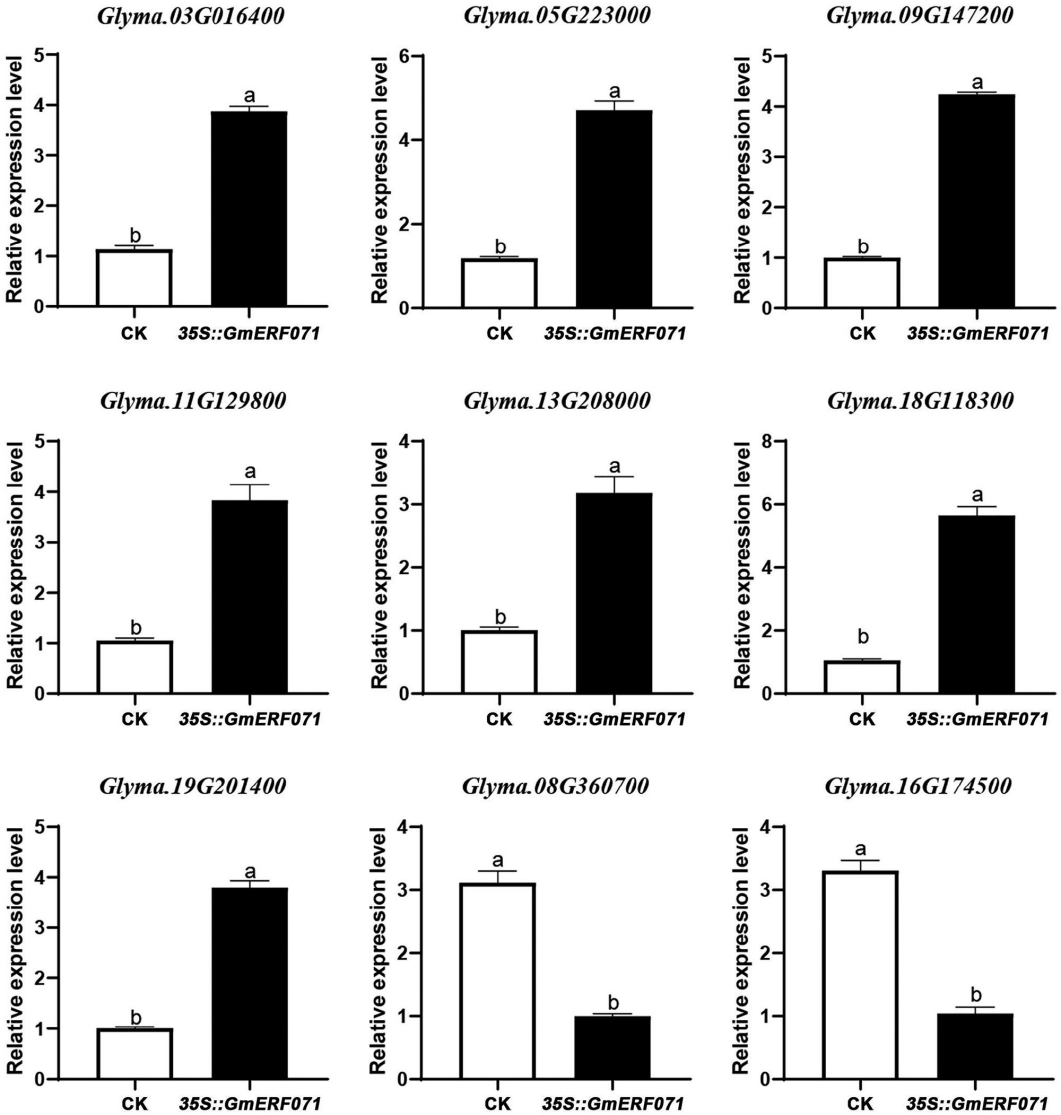
qRT‐PCR validation of nine differentially expressed genes in the *GmERF071* transgenic plant. CK is the Tianlong 1; 35S::*GmERF071* is the transgenic plant. Statistical difference was annotated with different letters (a and b).

After further screening with |log_2_FC| > 3, 49 candidate DEGs were identified between Tianlong 1 and *GmERF071* overexpression transgenic plants. In addition, nine related genes, *GlyMA.03G016400*, *Glyma.05G223000*, *Glyma.09G147200*, *Glyma.11G129800*, *Glyma.13G208000*, *Glyma.18G118300*, *Glyma.19G201400*, *Glyma.08G360700*, and *Glyma.16G174500*, which may be regulated by *GmERF071* against the SCN in soybeans, were selected, and their expression levels were determined to confirm the RNA‐seq results. The qRT‐PCR results showed that the expression of *Glyma.08G360700* and *Glyma.16G174500* decreased, whereas the expression of the other seven genes increased in transgenic soybean (Figure 7), which was consistent with the RNA‐seq results (Table S2).

## 4 DISCUSSION

SCN is a destructive pest in soybean fields worldwide (Kofsky et al., 2021). To provide novel insights into the molecular biology of soybean varieties resistant to SCN, transcriptomic analysis of PI88788 and Peking soybean showed that *GmERF071* encoding the ERF domain was upregulated in Peking‐ and PI88788‐type resistant sources (Chu et al., 2022). The mechanism of action of *GmERF071* in SCN resistance was verified in *GmERF071* overexpression transgenic plants (Figure 3). The KEGG pathway was the most enriched in plant‐pathogen interactions using transcriptome analysis. In addition, the cis‐acting elements within the promoter region of *GmERF071* have relevant acting elements involved in salt stress induction. However, the *GmERF071* transgenic and control plants showed no statistically significant differences under salt stress (Figure S4). These results suggest that *GmERF071* was involved in resistance to SCN, but not in salt stress.

*GmERF071* encodes a protein belonging to the adaptor‐related protein complex two family. Protein structure prediction revealed that the protein is a hydrophilic, unstable protein with an AP2/ERF family subfamily ERF domain. The protein encoded by *GmERF071* was localized in the nucleus (Figure 2), consistent with the nuclear localization of transcription factors. ERFs and RAP2.2, which encode an ERF transcription factor, play important roles in plant stress responses (Zhao et al., 2012). Plant hormones are involved in SCN resistance. Ethylene is important for plant resistance to necrotrophic pathogens, and ERFs are often indispensable (Phukan et al., 2017). A previous study reported that the soybean miR159‐*GmMYB33* module is directly involved in GA‐modulated soybean resistance to SCN (Lei et al., 2021). Genes related to the plant hormone signal transduction pathway were upregulated in *GmERF071* overexpression transgenic plants (Figure 7). In the RNA‐seq data, except for *GmERF071* (*Glyma.19G262700*, log_2_FC 1.85), a total of six genes of the ethylene‐responsive transcription factor (*Glyma.19G213100*, log_2_FC 1.43; *Glyma.20G195900*, log_2_FC 2.54; *Glyma.20G155200*, log_2_FC 1.89; *Glyma.02G080200*, log_2_FC 2.45; *Glyma.01G231000*, log_2_FC 2.62; *Glyma.01G225000*, log_2_FC 2.15) were changed, and all of them were upregulated (Table S2). The resistance mechanism of *GmERF071* to SCN may be mediated by triggering the expression of defense genes and mediating plant hormone signal transduction. During the host‐pathogen arms race, plants have evolved resistance against various pathogens via complicated resistance pathway responses. The MAPK signaling module, activated by nematode infection or wounding, is essential for the development of SCN resistance in soybeans. GmMPK3‐dependent and GmMPK6‐dependent phosphorylation of GmCDL1 improved GmMPK3 and GmMPK6 activation and soybean resistance against multiple diseases, respectively, illustrating a positive feedback mechanism (Zhang et al., 2024), which was consistent with the upregulated expression of MAPK pathway‐related genes in the *GmERF071* overexpression transgenic plant (Figure 7). *Glyma.19G201400* is associated with plant‐pathogen interactions involved in infection and pathogenicity (Gao et al., 2014). The expression of *Glyma.19G201400* increased in *GmERF071* overexpression transgenic soybean. The response to stimulus pathway enriched by the response to the stimulus gene of *Glyma.08G360700* was upregulated in *GmERF071* overexpression transgenic plants, which may be involved in pathogens, defense response, cell death, and hypersensitive response. Flavonoid compounds have strong antioxidant and antibacterial properties that regulate plant growth and developmental processes and improve plant adaptability and resistance (Akbar et al., 2023). Phenylpropionic acid biosynthesis is activated after pathogen attack in various plants (Kostyn et al., 2012; Li et al., 2024). *Glyma.09G147200* is activated in plants after SCN infection. *Glyma.08G360700* is involved in the plant hormone signal transduction pathway, and various signaling cascades involving different phytohormones regulate the soybean response to SCN (Hawk et al., 2024). GO terms of the polysaccharide catabolic process, response to carbohydrates, and pathways of starch and sucrose metabolism were enriched in the DEGs. Sucrose and carbohydrates are sugar sources for nematode‐induced syncytia (Hofmann & Grundler, 2008). Starch and sucrose metabolism play significant roles in maintaining normal plant growth, development, and defense‐related responses to biotic and environmental stresses (Chen et al., 2023; Jeandet et al., 2022). A previous study indicated that starch and sucrose metabolism pathways play a role in mediating the susceptible response to the *Heterodera glycines* race 5 (Huang et al., 2022). During nematode development and feeding, starch and polysaccharides are used to compensate for sugar variability (Ibrahim et al., 2011). *Glyma.11G129800* is associated with the biosynthesis of phenylpropane, which is related to disease resistance in plants (C. Li et al., 2024; Ojeda‐Rivera et al., 2024; Wang et al., 2024) and is involved in starch and sucrose metabolism.

A previous study indicated that many metabolic processes, including primary carbon metabolism such as amino acid synthesis and glycolysis, change after SCN infestation of soybean roots (Afzal et al., 2009). In addition, *Glyma.11G129800*, *Glyma.05G223000*, *Glyma.13G208000*, and *Glyma.19G201400* were associated with GO during metabolism. *Glyma.05G223000*, which is associated with cysteine and methionine metabolism, regulates the synthesis of 1‐aminocyclopropane‐1‐carboxylate synthase, which plays a key role in ethylene synthesis and phosphopyridoxal binding (Khan et al., 2024). *Glyma.13G208000*, which encodes an aspartyl protease, may be employed in the anti‐SCN process in soybeans by changing the cell structure and regulating programmed cell death (Wan et al., 2015). *Glyma.19G201400* encodes calcium‐dependent protein kinases involved in soybean‐insect interactions and respond to drought stress (Hettenhausen et al., 2016). Moreover, cyanuric acid metabolism is an intermediate of many metabolic pathways for amino acids and important biological molecules. Cyanuric acid is involved in the metabolic resistance of plants (Al‐Zahrani et al., 2022; Esquirol et al., 2020). Therefore, metabolism‐related processes are implicated in altering the resistance of soybeans promoted by *GmERF071* to SCN. Previous studies have demonstrated their direct and indirect effects on plant stress responses. *Glyma.09G147200* belongs to the DREB subfamily of the AP2/ERF family. They play an essential role in regulating the expression of stress‐related genes in response to abiotic stress (Mei et al., 2022). *Glyma.16G174500* is differentially expressed in the roots of the Rbs3‐resistant genotype of the fungus *Phialophora gregata* (Mccabe et al., 2018). These genes may be related to *GmERF071* in soybean SCN resistance regulation.

## 5 CONCLUSION

We identified a novel SCN resistance gene *GmERF071* and verified its molecular function by soybean genetic transformation and physiological analysis. Overexpression of *GmERF071* effectively enhanced the SCN resistance of soybeans. Seed length, seed width, and 100‐seed weight were significantly decreased in *GmERF071* transgenic plants, whereas oil content and protein content were not significantly affected. RNA‐seq results suggested that GmERF071 plays a key role in SCN resistance by regulating starch and sucrose metabolism, plant‐pathogen interactions, and plant hormone signal transduction pathways. In addition, some genes such as *Glyma.05G223000*, *GlyMA.09G147200*, *Glyma.11G129800*, *Glyma.13G208000*, *Glyma.19G201400*, *Glyma.08G360700*, and *Glyma.16G174500* may play important roles in the defense response of *GmERF071* in soybean to SCN. These results may offer novel perspectives for investigating candidate‐resistant varieties of SCN in soybeans.

## AUTHOR CONTRIBUTIONS

**Erhui Xiong**: Funding acquisition; investigation; validation; writing—original draft; writing—review and editing. **Jiaqi Xu**: Data curation; investigation; validation. **Pingzhang Feng**: Data curation; formal analysis; investigation; validation. **Yun Lian**: Conceptualization; funding acquisition; methodology; writing—review and editing. **Xiaoling Zhi**: Data curation; software; validation. **Ke Li**: Data curation; investigation; validation. **Erhan Zhang**: Validation. **Bing Li**: Resources; software; validation. **Shijie Zhao**: Resources; software. **Changzhong Liu**: Validation. **Chengyu Wei**: Resources; software. **Panpan Li**: Resources; validation. **Yaping Zhao**: Resources; software. **Lipei Zhao**: Software. **Mengwei Zheng**: Resources; software. **Heng Zhang**: Resources; software. **Yi Li**: Software; validation. **Shanshan Chu**: Conceptualization; funding acquisition; writing—review and editing. **Yongqing Jiao**: Conceptualization; data curation; formal analysis; funding acquisition; methodology; resources; writing—original draft; writing—review and editing.

## CONFLICT OF INTEREST STATEMENT

The authors declare no conflicts of interest.

## Supporting information




**Table S1 Primer sequences of qRT‐PCR**.

**Table S2 DEGs between Tianlong 1 and *GmERF071* overexpression transgenic plants**.

**Figure S2 Soybean cyst nematode (SCN) number of CK and 35S::*GmERF071* in hairy roots**. (A) Phenotype of CK and 35S::*GmERF071* hairy roots; (B) The SCN number in hairy roots between CK and 35S::GmERF071 lines.
**Figure S3 Penetration and development of SCN in Tianlong 1 and *GmERF071* transgenic lines after infecting cysts**.
**Figure S4 Salt resistance of *GmERF71* transgenic plants**. (A) Pre‐test of salt stress treatment. C1, C2, C3, C4, and C5 were 0, 50, 100, 150, and 200 mmol/L of salt solution treatment, and 1d, 2d, and 3d were 1, 2, and 3 days after treatment, respectively; Bar = 5 cm. (B) Phenotypic diagram of control and transgenic plants under 150 mmoL/L salt stress; Bar = 10 cm. CK was the Tianlong 1 control, and #1, #2, and #3 were the three positive strains of *GmERF71* transgenic plants. (C) Line comparison diagram of survival rate of control and transgenic plants under 150 mmoL/L salt stress.

## Data Availability

All data generated or analyzed during this study are included in this published article (and its supplementary information files).
