# Supplementary material for: Overexpression of the GmERF071 gene confers resistance to soybean cyst nematode in soybean
Source: Plant Genome. 2025 Apr 29;18(2):e70033. doi: 10.1002/tpg2.70033 (PMC12041739; doi:10.1002/tpg2.70033)
Supplement: Supplementary file 1 [file TPG2-18-e70033-s001.pdf]

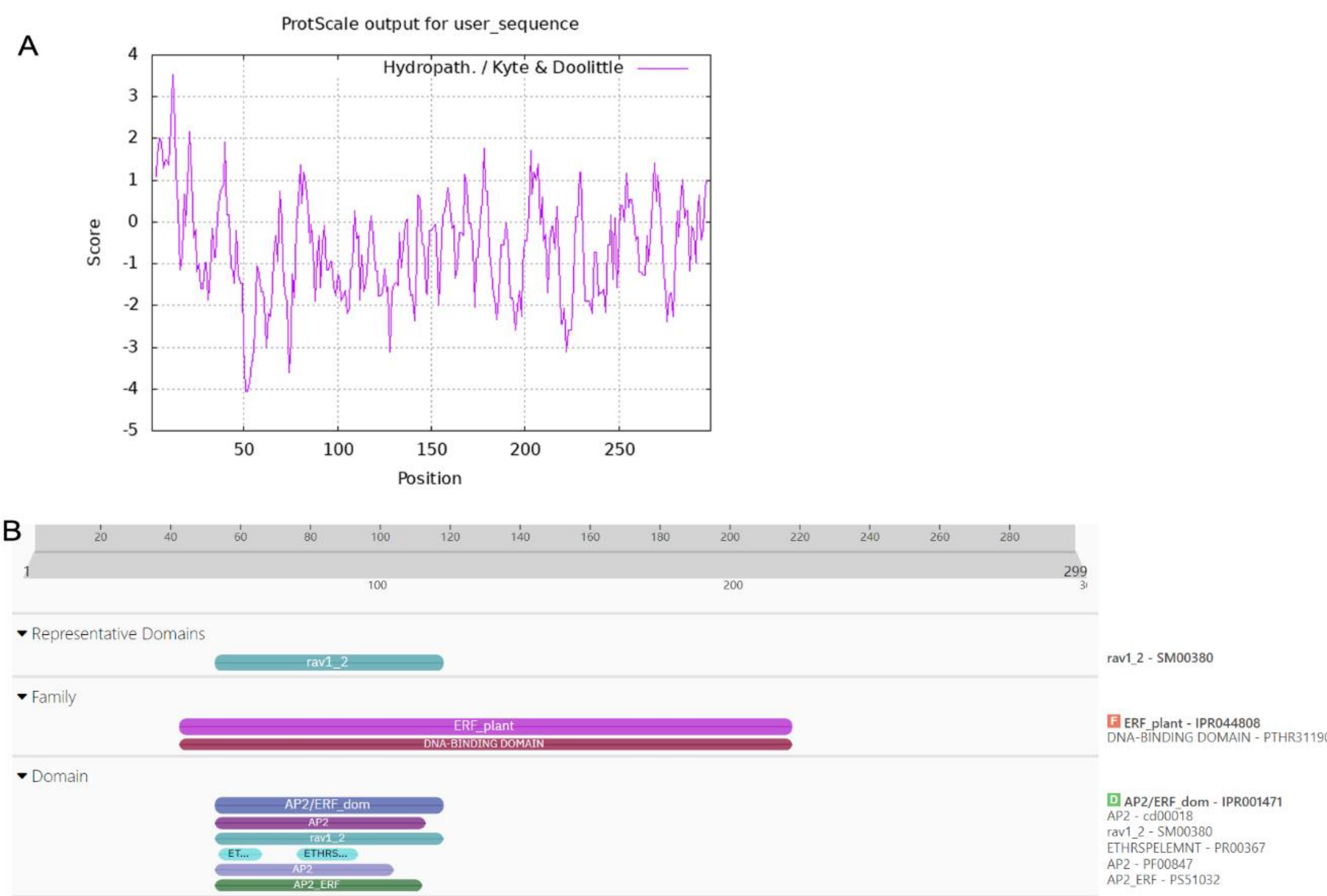
A
ProtScale output for user_sequence
Hydropath. / Kyte & Doolittle
Score
Position
4
3
2
1
0
-1
-2
-3
-4
-5
50
100
150
200
250
B
20
40
60
80
100
120
140
160
180
200
220
240
260
280
1
299
100
200
Representative Domains
rav1_2
rav1_2 - SM00380
Family
ERF_plant
DNA-BINDING DOMAIN
ERF_plant - IPR044808
DNA-BINDING DOMAIN - PTHR31190
Domain
AP2/ERF_dom
AP2
rav1_2
ET...
ETHRS...
AP2
AP2_ERF
AP2/ERF_dom - IPR001471
AP2 - cd00018
rav1_2 - SM00380
ETHRSPELEMNT - PR00367
AP2 - PF00847
AP2_ERF - PS51032

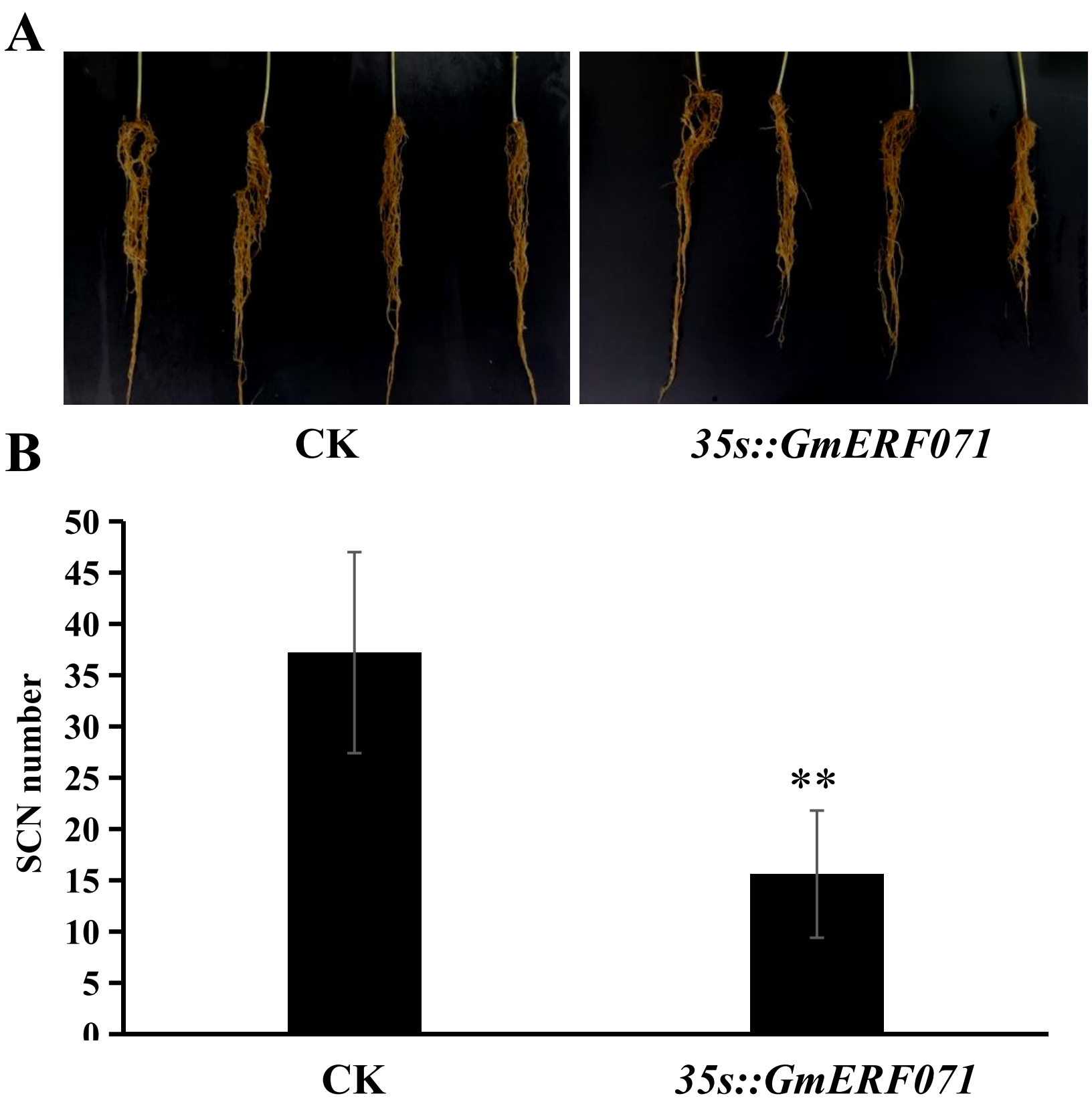
A
B
CK
35s::GmERF071
SCN number
50
45
40
35
30
25
20
15
10
5
0
**
CK
35s::GmERF071

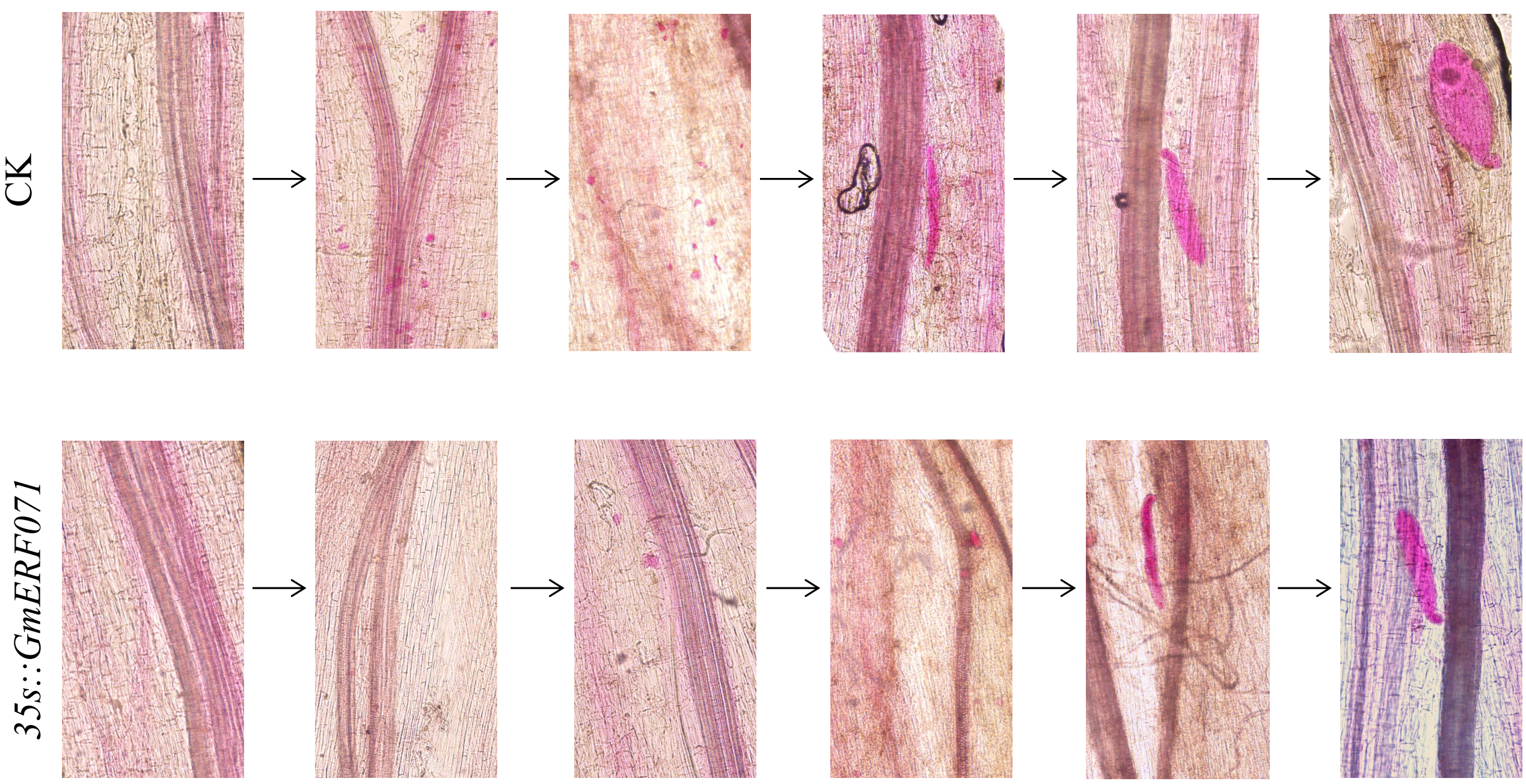
CK
35s::GmERF071

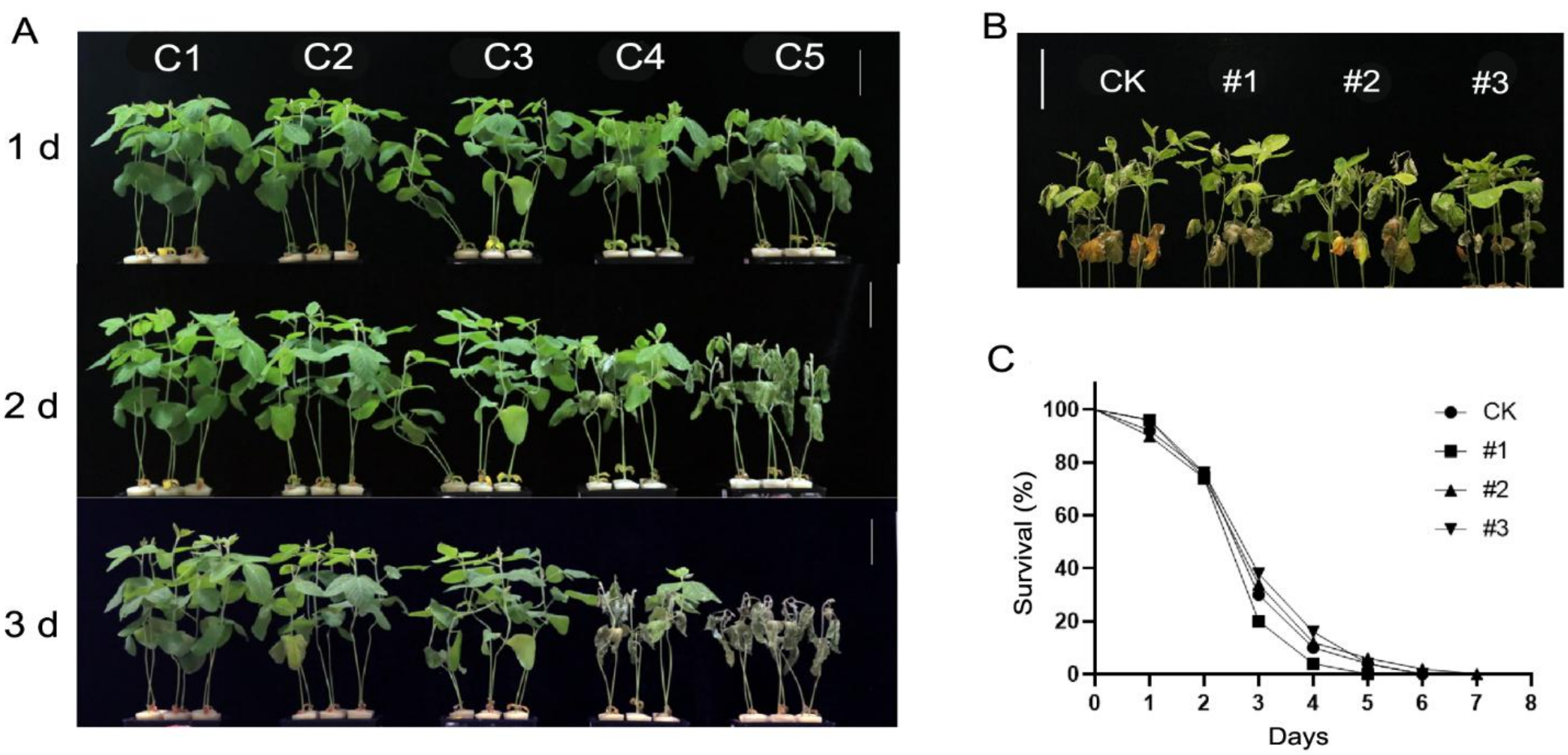

A
C1
C2
C3
C4
C5
1 d
2 d
3 d
B
CK
#1
#2
#3
C
Survival (%)
Days
CK
#1
#2
#3
