# Supplementary material for: Overexpression of the GmERF071 gene confers resistance to soybean cyst nematode in soybean
Source: Plant Genome. 2025 Apr 29;18(2):e70033. doi: 10.1002/tpg2.70033 (PMC12041739; doi:10.1002/tpg2.70033)
Supplement: Supplementary file 1 — Table S1 Primer sequences of qRT‐PCR. [file TPG2-18-e70033-s003.docx]

Table S1 The primer sequences of qRT-PCR

| Gene ID | Forward primer (5’→3’) | Reverse primer (5’→3’) |
| --- | --- | --- |
| *Glyma.18G290800* | CGGTGGTTCTATCTTGGCATC | GTCTTTCGCTTCAATAACCCTA |
| *Glyma.03G016400* | TAACAGGTTCAAGGGCTCCCG | CGTCGTCTTCAACGATGCG |
| *Glyma.05G223000* | CAGCGAGAAGGATGTCGAGTT | ACTCTATGCCAACACTTTCCA |
| *Glyma.09G147200* | TGCTTCCCTCACTTCCCTTTA | ACTGGAGTGAGTAGTGTTGTGG |
| *Glyma.11G129800* | TGGTTTTTGCCGCTCAGAGA | TCGCTTAGATCAGGTGCGTC |
| *Glyma.13G208000* | CCAATTCAGCTTGCATCGGC | TTGAAGGAACACCCTCCACG |
| *Glyma.18G118300* | AGACCCTAGCAAGTCCACCA | TCTCAACTCAGGAGTGCTGC |
| *Glyma.19G201400* | GAAACCAGTTCCACTTCCAACC | CATATTGCCCCTTCCCAAGC |
| *Glyma.08G360700* | TCAAAACCGAGTACGCAAGC | GCAGAGGGAGAGTGTTGGTA |
| *Glyma.16G174500* | GATGACGTCTCCACGAGCAT | TTGCAGCGAGAATCTGGCAC |
| *Glyma.19G262700* | CTGTTGCCTTCTGGAATGGT | AGCAGCCAAGATTGTTTCACA |
