# Supplementary figures and images for: Overexpression of the GmERF071 gene confers resistance to soybean cyst nematode in soybean
Source: Plant Genome. 2025 Apr 29;18(2):e70033. doi: 10.1002/tpg2.70033 (PMC12041739; doi:10.1002/tpg2.70033)

A

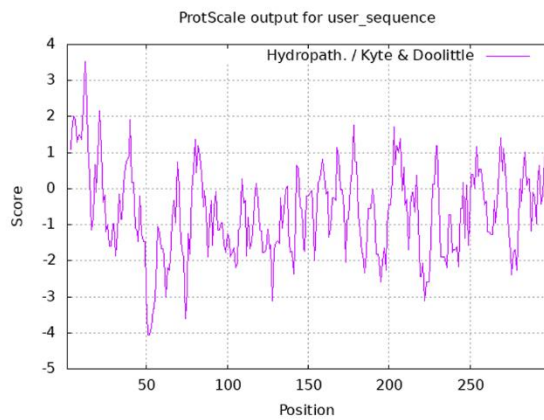

B

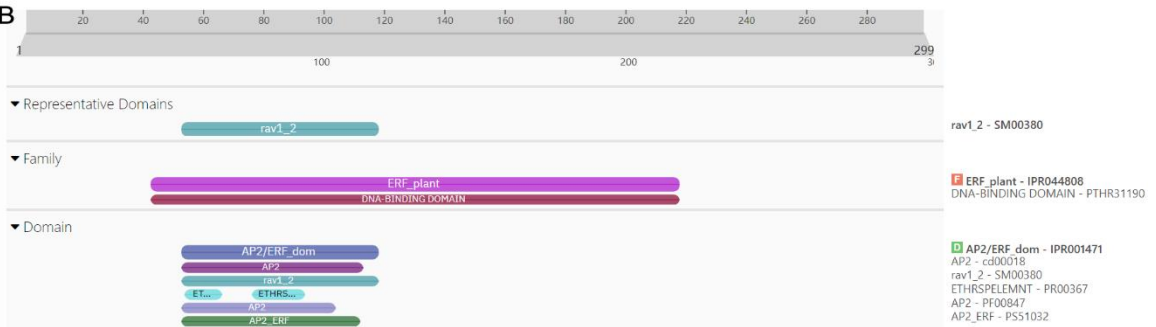

**A**

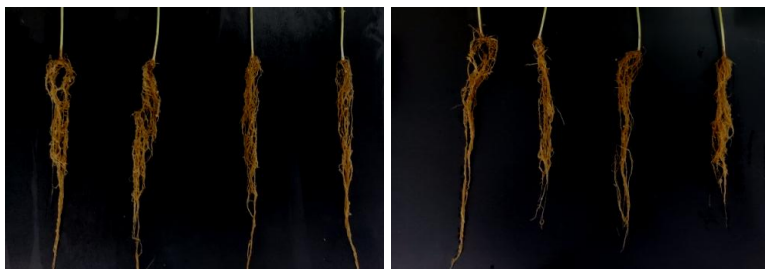

**B**

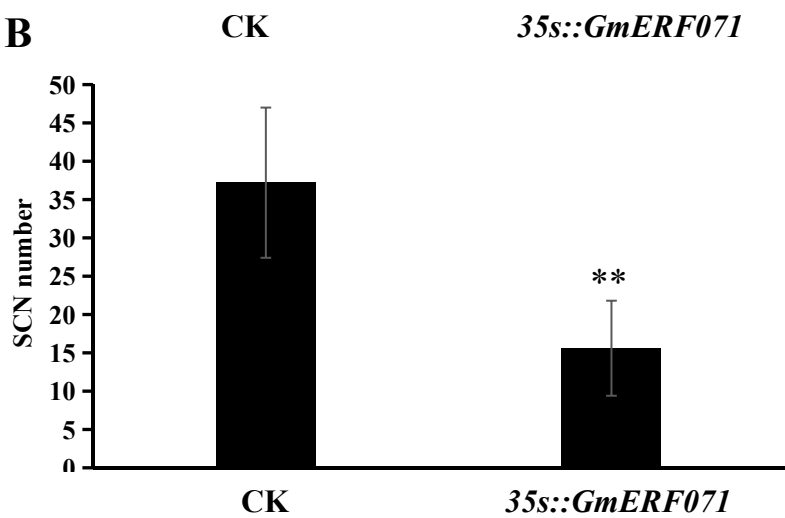

CK

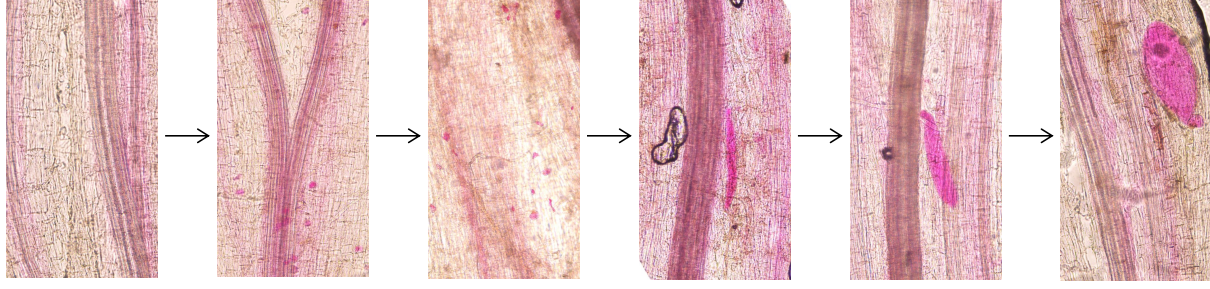

*35s::GmERF071*

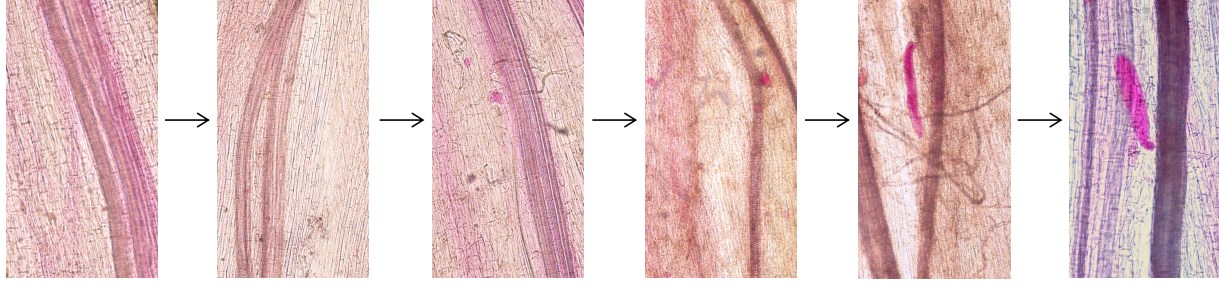

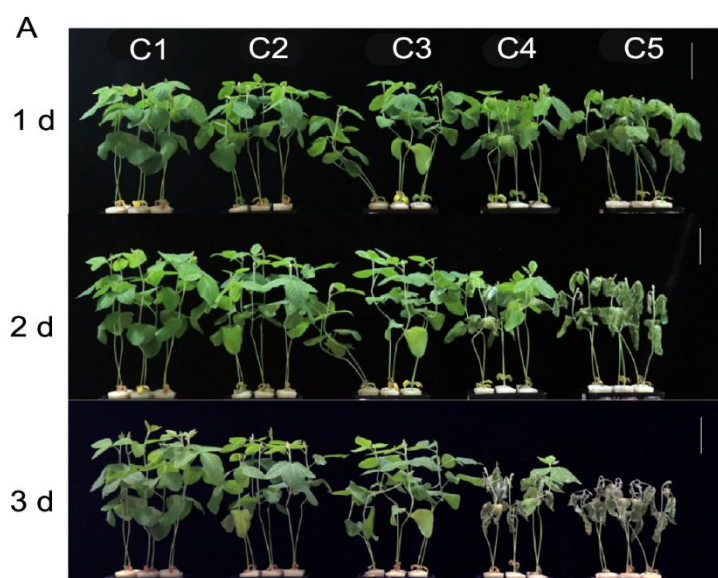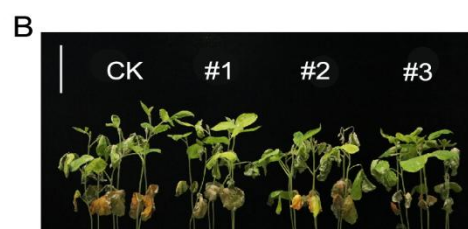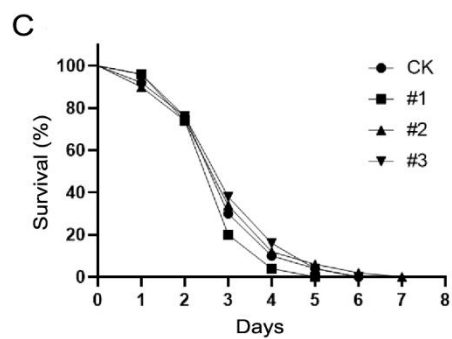

Supplement: Supplementary file 3 — Figure S2 Soybean cyst nematode (SCN) number of CK and 35S::GmERF071 in hairy roots. (A) Phenotype of CK and 35S::GmERF071 hairy roots; (B) The SCN number in hairy roots between CK and 35S::GmERF071 lines. Figure S3 Penetration and development of SCN in Tianlong 1 and GmERF071 transgenic lines after infecting cysts. Figure S4 Salt resistance of GmERF71 transgenic plants. (A) Pre‐test of salt stress treatment. C1, C2, C3, C4, and C5 were 0, 50, 100, 150, and 200 mmol/L of salt solution treatment, and 1d, 2d, and 3d were 1, 2, and 3 days after treatment, respectively; Bar = 5 cm. (B) Phenotypic diagram of control and transgenic plants under 150 mmoL/L salt stress; Bar = 10 cm. CK was the Tianlong 1 control, and #1, #2, and #3 were the three positive strains of GmERF71 transgenic plants. (C) Line comparison diagram of survival rate of control and transgenic plants under 150 mmoL/L salt stress. [file TPG2-18-e70033-s001.pdf]
